# Inquiries into the Frequency of Hernia, According to Sex and Age, and in Relation to Population

**Published:** 1844-04

**Authors:** 


### REVIEWS.

---

Art III.—*Recherches sur la Fréquence des Hernies, selon les Sexes, les Ages, et relativement à la Population.* Par J. F. Malgaigne ,Professeur agrégé de la Faculté de Medicine de Paris, Chirurgien de l'Hospice de Bicêtre.

*Inquiries into the Frequency of Hernia, according to Sex and Age, and in relation to Population.* By J. F. Malgaigne, &c. From the *Annales d'Hygiéne Publique et de Médecine Légale*, Vol. xxiv, 1840.

Accident having directed our attention to this article a few days ago, a hasty perusal of it led us to believe that an analysis would be acceptable to the readers of the Journal. The paper being in itself an analytical one, the subject will thus have undergone a sort of double distillation after passing through our alembic, and hence to readers of *refined* taste must prove agreeable. At all events, as remarked by the author, if the question considered here be not one of that kind which leads to important practical results, it still offers so much interest that most writers upon hernia have attempted its solution.

The data upon which some of the writers upon this subject have founded their conclusions, are exceedingly vague; and the results obtained by others, who have attempted to solve the problem by figures, are always doubtful, and indeed generally contradictory. Arnauld, for instance, says that of males, at least one-eighth are affected with hernia, but he offers no proof of it; others have carried the proportion to one-eighth of the total population. Bordenave opposes strongly such an exaggeration, and fearlessly asserts that the proportion does not amount to one in a hundred. Louis, not content with assertions, made inquiries at the hospitals Saltpétriére, Bicêtre, and Pitié, and that for Invalids, and found that children were affected in the proportion of 1-50th, women 1:32, adults and old men 1-17th to 1-18th; results as different from the assertion of Bordenave as from that of Arnauld. Juville advances the idea that in Germany and the north of Europe, the proportion for men is 1-30th and more; in Italy and Spain, 1-15th; in France and England, more than 1-20th. Turnbull, in England, says, on the authority of researches carefully made in all the kingdom, that the individuals affected with hernia constitute 1-15th of the population. Lawrence thinks the statement manifestly exaggerated. Sheldrake goes still further, and makes the proportion 1-10th.

Knox, some years since, discussed this question in a special work, and although he has not expressed his opinion clearly, it is evident that he regards hernia as infinitely more rare than most authors do, and coincides very nearly with Bordenave. He rests his conviction on the following facts: 1. The statement in Monroe's work of the examination of recruits for the German legion by Versturme and others, which gives less than 1 in the 100. 2. Examination of 6229 recruits, by Marshall, from December, 1824, to December, 1825, giving a proportion of 1 in 76. 3. Two tables of recruits examined at the Dublin depôt—one of 4012 individuals, which furnished 18 ruptures, another of 2588 which gave 16; these tables united with that of Marshall, show that subjects with rupture do not form 1-100th of the total number. 4. Eighty-six individuals carefully examined after death, by himself, and only one with rupture found. 5. The last and principal argument is drawn from results of the draughts for the French army in 1831, '32 and '33, which show for the whole number 1.3 for every 100.

Finally, Marshall himself, makes a computation from the register of recruits at Dublin for the British army: the first table embraces 20 years, and gives a proportion of 1:50; a second embracing 23 years, of 1:48.

These calculations and results, besides being contradictory, are radically defective. They refer to a peculiar population, chosen from hospitals or the army, which must exhibit different proportions from the ordinary population; nor are the differences in the frequency of hernia, arising from sex and age, taken into consideration.

"The preliminary conditions, then, to be fulfilled in order to solve this difficult problem are: 1. To act upon the ordinary population without distinction and without choice. 2. To find the precise ratio of hernia in the two sexes. 3. To seek the different proportions of hernia at the different periods of life, and from all these combined, to deduce the general ratio."

The *bureau central* of the Parisian hospitals, Malgaigne says, fills the first condition, inasmuch as individuals of both sexes and of all ages, indiscriminately, apply there for trusses. But it may be objected to observations collected here, that they relate to paupers, or to a population bordering on indigence, and that it is not yet ascertained that the same law of production obtains among the wealthier classes. Laying aside, for the time, this question, the importance of which is acknowledged, and supposing that the persons submitted to his observation represent the entire population, he endeavors to establish

1. *The comparative frequency of hernia in the two sexes.*—The first tables of a suitable character to determine this question, are those of Louis, already referred to, from which it may almost be concluded that there are two males affected with hernia for one female, "a proportion truly extraordinary." These ruptures, however, were observed chiefly among the aged, and there are in fact, as will be seen presently, certain periods of life in which the frequency of hernia among females, brings the proportion pretty near that just stated.

But taking the entire population, as do the bandagists and chiefs of public establishments, the number of males is seen to increase considerably, and that of females to diminish. Monnikoff of Amsterdam has drawn up a table, which gives a proportion nearly of three males to one female (2.81 to 1); Mathey, of Anvers, another which gives 4 to 1; the statement of the old London Truss Society (up to 1814*) as published by Lawrence, furnishes about 6 to 1, the new society a little more than 6 to 1. There is nothing to show whether these tables have have been properly sifted; whether, for example, care has been taken to remove all those who were served twice, a thing which happens more frequently with men than women, as the latter do not wear out their trusses so quickly. Knox, moreover, observes that men submit more willingly to an examination, an important circumstance, especially in England, and one which must alter the real ratio of hernia in the two sexes.

As this last source of error scarcely exists at Paris, among the poor especially, the true proportion may be more easily obtained. The first observations collected by Malgaigne at the bureau central (October and November, 1835) amounted to 410 only: 335 males, 75 females; showing a proportion of 4½ to 1, which is the mean between that of Monnikoff and the English writers, and approaches the result obtained by Mathey of Anvers. But this number appearing too small to decide the question, especially in comparison with the numbers of Monnikoff and the London Societies, he collected the total number that presented themselves, at the same place, in 1836,

* In the last (5th) edition of Lawrence's Treatise, this report is brought up to 1835. The society, since it was first instituted, a period of 28 years, had afforded relief to 83.584 persons, of whom 67.798 were males, and 15.786 females. But this includes numerous cases of anal, uterine, vaginal and vesical prolapse. Subtracting these leaves

| | |
|---|---|
| Whole number, | 80.492. |
| Males, | 67.346. |
| Females, | 13.146. |

A proportion of about 5 (5.12) to 1. C.

and eliminating all who had applied twice and thrice, and all of the females who applied for pessaries only, found the whole number to be 2,767; *i. e.* 2,203 males, 564 females, giving a proportion something less than 4 (3.91) to 1. He repeated the same thing for 1837, and eliminating as before the cases of vaginal prolapse, and taking care to include none of the cases of the year preceding, found a total of 2,373, or 1,884 males, 489 females, a proportion still a little less than 4 to 1 (3.89 to 1.)

"This second result approaches so near the first that chance doubtless comes in for something; and it is not probable that a third series would give a proportion differing 0.02 only from the preceding. But we may at least presume that it would not deviate very greatly from it; so that we have sufficient reasons to conclude, that for Paris at least, and among the lower classes, about 4 subjects with hernia may be counted among males for 1 among females."

2. Our author next considers *the comparative frequency of hernia according to age.*

The only document bearing upon this branch of the subject, is that emanating from the Old London Truss Society, given by Lawrence, and to which reference has already been made. This presents some striking analogies with the tables subsequently given by our author, but is liable to so many objections on the score of accuracy that he makes no use of it. The 410 observations collected by himself during two months of 1835, after being disposed according to sex and age, furnish so small a number for each period of life that he attaches but little importance to it; this table we shall omit, although the author occasionally refers to it. But in the following table we have the cases collected in 1836 and 1837, arranged according to sex and age; this he thinks "worthy to fix the attention," and upon it, "he relies with some confidence."

| AGE OF SUBJECTS. | 1836. | | | 1837. | | |
|---|---|---|---|---|---|---|
| | MALES | FEMALES. | TOTAL. | MALES | FEMALES. | TOTAL. |
| From birth to 1 year. | 48 | 7 | 55 | 30 | 8 | 38 |
| From 1 to 2 years. | 16 | 7 | 23 | 29 | 7 | 36 |
| 2 " | 18 | 4 | 22 | 14 | 2 | 18 |
| 3 " | 11 | 4 | 15 | 9 | 1 | 10 |
| 4 " | 9 | 0 | 9 | 9 | 4 | 13 |
| From 5 to 13 " | 53 | 12 | 65 | 45 | 6 | 51 |
| " 13 to 20 " | 83 | 8 | 91 | 76 | 7 | 83 |
| " 20 to 28 " | 118 | 17 | 135 | 108 | 23 | 131 |
| " 28 to 30 " | 47 | 9 | 56 | 33 | 12 | 45 |
| " 30 to 35 " | 119 | 26 | 145 | 112 | 28 | 130 |
| " 35 to 40 " | 237 | 53 | 290 | 181 | 47 | 228 |
| " 40 to 50 " | 410 | 131 | 541 | 312 | 111 | 423 |
| " 50 to 60 " | 457 | 118 | 575 | 385 | 98 | 483 |
| " 60 to 70 " | 367 | 119 | 486 | 324 | 100 | 424 |
| " 70 to 75 " | 133 | 31 | 164 | 132 | 18 | 150 |
| " 75 to 80 " | 62 | 13 | 75 | 65 | 15 | 80 |
| " 80 and upwards. | 15 | 5 | 20 | 20 | 2 | 22 |

We must now let the author speak for himself; the dependence becomes too close for analysis:

"I have taken care to distribute the cases of hernia according to the periods that appeared to offer marked differences with those preceding and those following. Above all, it was interesting to know in what proportions the subjects with rupture occurred during the first year. In 1836, I find 55 out of 2,767, or 1:50; in 1837, 38 out of 2,373, or 1:62; mean term 1:56. If we confine ourselves to the males, the proportion will be, for the former year, 1:46, for the second, 1:62, as for the two sexes united; mean term 1:54.

"In children from one to two years of age, the number falls perceptibly for our first series; it continues almost the same for the second. But for the succeeding epochs of 2, 3 and 4 years, the diminution is flagrant and unquestionable; still, to the mass furnished by the first year, add those produced during the second, third and fourth; and if it be true that death decimates largely the population at these ages, it by no means produces such a diminution as we see occurring in this small *hernious* population. Do the radical cures thus reduce the number? These cures are not so common, or so rapid, especially with the trusses furnished by public charities, badly applied by their officers, and worse still by the parents. Is the mortality greater among subjects with hernia than among others? The question is one of high importance both in reference to practical surgery and public hygien, but which cannot now be answered.

"In the eight succeeding years, from 5 to 13, the decrease continues: the mean for each year is 8 for 1836, 6 for 1837, a remarkable agreement, which will be found, moreover, for nearly every period. It seems also that in these eight years, it is the 8th or 9th year especially which holds the middle place, presenting the least number of ruptures, and constituting a limit between the hernias of infancy, which to that point decrease, and those of youth which then begin to increase. I have taken the numbers for each year of 1836 only, and find:

| | | | |
|---|---|---|---|
| At 5 years, | 8 boys, | 0 girls, | total 8 |
| 6 | 6 | 2 | 8 |
| 7 | 10 | 1 | 11 |
| 8 | 1 | 1 | 2 |
| 9 | 7 | 2 | 9 |
| 10 | 9 | 3 | 12 |
| 11 | 7 | 2 | 9 |
| 12 | 5 | 1 | 6 |

"Taking the boys or the girls, or the two together, still the mean of the four latter years will exceed that of the four former, which seems to indicate an increase (*recrudescence*) beginning at the 9th year.

"This increase is far more striking from 13 to 20, a period of seven years, which gives for each year a mean of 13 ruptures for 1836, and 12 for 1837. It is remarkable, however, that the increase is solely among the males. Among the females, the number remains stationary, or, if reliance may be placed on such small numbers, it even seems to diminish. The period of puberty, then, in males, is accompanied by a predisposition to hernia that is foreign to the other sex. Very probably this proceeds from the difference in the occupations embraced by boys and girls; but this belongs to the question of causes. From 13 to 20 the number of individuals with rupture continues nearly the same, offering no very evident progression.

"From 20 to 28, the number evidently augments, whether the individuals are considered in mass, or distinguished according to the sex affected. The annual mean, in 1836, rises to 17, and is not much less in 1837. Among the males there is an increase of a fourth upon the preceding period; among the females, of more than double. Here we can aattribute nothing to occupation; but marriage and pregnancy bring their powerful influence; and, we may remark in passing, it is after the age of 20 that prolapsus of the vagina and crural hernia, previously so rare, make their appearance in females."

No decided progression occurs in any of the years of this new period. But from 28 to 30 there is a remarkable increase, especially among the females. The general mean for each of the two years, is 28 in 1836, 22½ in 1837, and among the females it is double that of the preceding years.

The 10 years from 30 to 40 must be divided into two periods. During the first five years the general mean is 29 for 1836, 26 for 1837, an increase, though slight compared with the extraordinary augmentation from 35 to 40. In the latter period, the mean of each year is double that of the preceding, both for males and females. The transition is abrupt. The agreement between the two tables is remarkable, and the more so because the mean of each of these five years is not only superior to the preceding, but remains superior to the succeeding. In fact, from 40 to 50 the mean falls from 58 to 54 for 1836, from 46 to 42 for 1837; at the same time, however, a difference is perceived in the ratio of hernia affecting the two sexes. The hernia of females which, from having been one-fourth of that of males, became so rare as scarcely to reach this proportion until after the 28th year, seems towards 40 to be acted upon by new causes which carry the proportion beyond that of the first year.

"Take, for example, the two preceding periods, adding the two years together:

| | | |
|---|---|---|
| From 30 to 35, | 54 women, | 231 men. |
| 35 to 40, | 100 " | 418 " |

"The proportion of one-fourth is not quite reached. Compare this with the number in the two tables for the period of from 40 to 50 years.

| | | |
|---|---|---|
| For 1836, | 131 women, | 410 men. |
| 1837, | 111 " | 312 " |
| Total, - | 242 " | 722 " |

"The proportion of hernia among females is now one-third. Moreover, although from 40 to 50 the general mean for each year has really diminished, it has augmented for the females, and the proportion for each sex is as follows:

| | | | |
|---|---|---|---|
| From 35 to 40 years, annual mean: | 20 females, | 84 males. |
| 40 to 50 " " " | 24 " | 72 " |

"Is such a difference to be regarded as the work of hazard? The coincidence of the number for the two years forbids the supposition. To what new cause, then, must be attributed this sudden advance of hernia among females?"

From 50 to 60 there is an increase, and the annual mean becomes equal or even superior to what it was from 35 to 40. The ratio between the sexes is likewise nearly as in the latter period; whence it follows that from 50 to 60 there is an increase of hernia among males, and a diminution on the contrary among females, the latter forming scarcely more than a fourth: 118 to 457, 98 to 385.

From 60 to 70 the general sum falls; and that of females remaining the same, the ratio of the two sexes becomes almost 1 to 3, as in the period from 40 to 50.

"If we take in mass the decennial period from 70 to 80, we shall find that the amount of hernia among males has decreased not quite one-half, whilst among females it has fallen off two-thirds. At this age, spontaneous cures cannot be alleged; is the mortality, which we shall show to be greater among subjects with rupture than among the ordinary population, greater still among females affected with hernia than males?

"Again, I have divided these ten years into two equal periods, in order to show the rapid decline of the population affected with rupture. Each year thins the number; previously, the new cases that occurred filled up the vacancies made by death; from 60 to 70, for instance, the amounts for each year still balanced; but mark the progressive decrease beginning with the 70th year:

| | | | |
|---|---|---|---|
| At 70 years, | 38 men, | 10 women, | total 48 |
| 71 " | 29 " | 8 " | " 37 |
| 72 " | 30 " | 5 " | " 35 |
| 73 " | 17 " | 4 " | " 21 |
| 74 " | 19 " | 4 " | " 23 |
| 75 " | 20 " | 2 " | " 22 |
| 76 " | 15 " | 3 " | " 18 |
| 77 " | 8 " | 3 " | " 11 |
| 78 " | 13 " | 2 " | " 15 |
| 79 " | 7 " | 3 " | " 10 |
| 80 " | 4 " | 2 " | " 6 |
| 81 " | 3 " | 2 " | " 5 |
| 82 " | 3 " | — " | " 3 |
| 83 " | 1 " | — " | " 1 |

"After which I find but one subject with hernia for each of the three succeeding years; than a female aged 88, and lastly a man aged 98.

"To resume, the space comprised between birth and extreme old age, is found divided into a certain number of periods in which the frequency of hernia varies greatly, and for each of which it is necessary to compare the number of individuals affected to the total correspondent population. Evidently to take the ratio of hernia to the population at a determined age, and deduce therefrom a general conclusion, would be altogether erroneous; yet this is precisely what Knox has done. Supposing this ratio to be known, it would be necessary to establish an arithmetical proportion between the amount of hernia at this age and the total amount. If, relying on the results furnished by the years 1836 and 1837, we seek, for example, the proportion of hernia in the period from 20 to 28 to the general mass, we shall find for the male sex:

In 1836, 118 to 2,203, or 1: 18.67.
In 1837, 108 to 1,884, or 1: 17.40.

"The mean will be 1 to 18. If we wish to know then the relation of a single year of this epoch to all periods of life, we have only to take the eighth of the numbers 118 and 108 and compare it to the general numbers 2,203 and 1,884, or to perform any other equivalent arithmetical operation, and we shall arrive at this conclusion, that the sum of hernia furnished by a male population from 20 to 21, for example, is to the general sum of hernia in the entire male population as 1 is to 144."

3d. A more difficult question now presents itself, namely: *The frequency of hernia in the male population of* 20 *or* 21 *years.*

Our author chooses this period because it is the only one upon which data at all satisfactory can be obtained. To recruit the army in France, the law requires an exact census of the male population at this age to be taken annually. A general examination is made by a board of surgeons, and hernia being a cause of exemption, it would seem easy to find the relation it bears to a given number of individuals. But there are difficulties in the way. From the general mass thus registered, the law calls a certain number only, and when the council of revision have obtained the legal number of healthy young soldiers, the remainder are sent back without examination. It is here that Knox erred. He acted upon the entire registration for three successive years, 1831, '32, '33, and found the proportion to be 1. 3 for every 100; forgetting, or not knowing that the law required of each census, only 80,000 men. Instead, therefore, of taking the amount of the whole class, we must seek that of the individuals examined. This is composed of those recognised as fit for service, and of those exempted. From these latter must be subtracted still those that are exempted by virtue of the law itself, such as the elder son of widows, &c. After all this difficulties yet remain, as will presently be seen.

The documents employed by the author are all of an official character. Some of them were found in a work published by Chabrol in 1826, entitled: "*Recherches statistiques sur la ville de Paris et le département de la Seine;*" others were obtained from the Minister of War, and consist of reports made by the administration for six years from 1831 to 1837.

The *Recherches* of Chabrol set forth the results of the old conscription in the department of the Seine, from the year IX (of the Republic) to 1810, also those of the recruiting service from 1816 to 1823. In the first period, only 30,603 men were examined out of a male population (from 20 to 21) of 44,543;

In the second period, 11,730 only out of 40,576. The difference is enormous, but is to be explained by the difference in the contingent demands.

"Confining ourselves now to the subjects really passed to examination, and to the ruptures found among these, we find for the old conscription:

| | | | | |
|---|---|---|---|---|
| At Paris | 25,503 exam'd, | 694 hern., | 1 out of 36.74. |
| In the liberties (*banlieue*) | 5,100 " | 140 " | 1 out of 36.41. |

"From 1816 to 1823, separating in like manner Paris from its liberties, we find:

| | | | |
|---|---|---|---|
| At Paris | 9,836 exam'd, | 261 hern., | 1 out of 37.69. |
| In the liberties | 1,894 " | 52 " | 1 out of 35.73. |

"These different proportions present a remarkable agreement, and the general mean, 1 to 36.87, seems therefore to acquire a high degree of probability."

But here new difficulties arise. It might be supposed that all those rejected for slighter causes than hernia, had been carefully examined as to the existence of the latter, that the less cause was only admitted in the absence of a greater. But the law exempts individuals who have lost an eye, who are blind, lame, maimed, &c.; and these circumstances only are noted, although other causes may exist. Now are the blind, lame, &c. unaffected with hernia? An individual rejected for defect of stature, is not examined as to the existence or non-existence of a hernia. It is indispensably necessary, then, to throw out of from the table of examinations, those sent away on account of stature, or for graver causes. This destroys the previous calculation; the ratio of 1:37 becomes too small, nor is it possible precisely to determine it. Thus, subtracting those only rejected on account of small stature, and the proportions before given become,

| | |
|---|---|
| For the old conscription | about 1: 31.27. |
| For the new | about 1: 32.63. |

By pursuing this process in reference to more important infirmities than hernia, the ratio augments frightfully. But it is exceedingly difficult to determine, in many cases, from the tables, whether such an affection as scall, for example, served for the discharge of an individual laboring under a slight hernia, or whether a voluminous hernia attracted attention rather than a slight degree of scall. As hernia, moreover, is less common (according to Malgaigne) among men of small stature, the proportions, bearing solely on those of the required height, would of course be exaggerated.

"All these things being duly considered, it appeared to me that by compensating for this rarity of hernia, among subjects rejected for defect of height, with the surplus of hernia not mentioned, though probably existing among subjects rejected for graver causes, I might keep within the true limits, equally removed from exaggeration in *plus* or in *minus*; and consequently, from the numbers thus far mentioned, the proportion would be 1: 31.64, and more fully 1: 32."

The documents obtained from the War Department bear upon an imposing mass of individuals, and as they extend over a space of six years, the results will go far to destroy or confirm the inference just deduced from previous documents. Before presenting the table some explanation is necessary:

"The second column indicates the force of the class, the third the number called before the councils of revision. In reality, from this third column should be deducted the fourth, which comprises the individuals called in the order of their numbers, and who, not having answered, are set down as valid and declared soldiers. It must not be imagined that these refractory individuals are all fit for service, and neglect a proceeding which they know very well at the time would be useless. The Minister of War marks, on the contrary, their absence as punishable, because it causes to be directed upon regiments recruits for the most part infirm. The fifth column comprises two categories, first the legal exemptions which have to be submitted to the board and by them verified, but which render a coporeal examination useless; second, what is called the *deficit*, that is to say, individuals dying, or convicted of infamous crimes, after the period of enrollment, and who consequently have not been submitted to examination. This fifth column, therefore, must be deducted from the list of individuals called; the remainder comprises the valid subjects, the infirm, and those rejected for defect of height. I have not noted the valid subjects, the number of which, always fixed by law, is 80,000. I have also left out those rejected for want of height, as this element no longer interests us, and besides may be obtained from our tables by a simple arithmetical operation.

Finally the last two columns constitute the summary of each series, namely: the last but one, the exact number of individuals examined in relation to infirmities, valid or non-valid; and the last, the number of ruptures detected by the board of examination.

I thought it right to give all these numbers, more especially in order to place the finger, so to speak, upon the error committed by Knox, in not having decomposed, as I have, the quantities which express the total force of the class.

| Years. | Force of the class | Number called | Absent. | Legal exemptions & deficit. | Rejected for infirmities. | Total number examined. | Ruptures. |
|---|---|---|---|---|---|---|---|
| 1831 | 295.978 | 171.541 | 2.246 | 28.039 | 47.531 | 125.285 | 4.044 |
| 1832 | 277.477 | 166.305 | 2 136 | 27.263 | 43.908 | 121.772 | 3.579 |
| 1833 | 285.805 | 172 397 | 3.572 | 29.128 | 48.175 | 124.603 | 4.222 |
| 1834 | 326.298 | 171.772 | 2.545 | 28.990 | 48.316 | 125.771 | 3.994 |
| 1835 | 309.376 | 173.765 | 2.357 | 30.316 | 49.009 | 126.652 | 3.921 |
| 1836 | 309.516 | 179.317 | 2.398 | 31.147 | 53.190 | 130.792 | 4.461 |

"Seek now the exact proportion of subjects with hernia in those examined, and you have the following results:

| | | |
|---|---|---|
| In | 1831 . . . . . . . . . . . . | 1: 30.98 |
| | 1832 . . . . . . . . . . . . | 1: 34.05 |
| | 1833 . . . . . . . . . . . . | 1: 29.95 |
| | 1834 . . . . . . . . . . . . | 1: 31.49 |
| | 1835 . . . . . . . . . . . . | 1: 32.30 |
| | 1836 . . . . . . . . . . . . | 1: 29.34 |

"And by adding the several sums together, you will obtain 24,221 persons with rupture in a total mass of 754,875 passed to examination; the proportion is a little more than 1:31, rigorously 1: 31.16. This is a remarkable conformity with the proportion deduced from the documents furnished by the department of the Seine; and it appears to me sufficiently demonstrated that the general proportion is from 1:31 to 1:32. I myself hold to the latter for reasons already explained, and if any one finds it too small, it will be easy for him to rectify at his pleasure the calculations I am now about to enter upon."

4. The next inquiry is, *The general ratio of ruptures to the sum total of the population.* As this section is short we present it without abridgement.

"The frequency of ruptures at the age of 20 being known, it is easy to arrive at their proportion for all ages. Thus I take table III of the *Annuaire des Longitudes*, exhibiting the law of population in France for ten millions of inhabitants, the population from 20 to 21 years comprises 173,576 individuals, or for the males 86,788. Of these the proportion with ruptures at 1:32 will be 2,712

"On the other hand, if we seek in each of our two tables what is the exact number at the age of 20—21 for males, we find it by taking the eighth of the amount assigned to the period of eight years from 20 to 28. This eighth, for the first table, 1836, is equal to 14.75 ruptures; for the second, 1837, to 13.50 ruptures.

"Comparing these quantities to the sum total of each year, it is found that the ruptures observed in subjects from 20 to 21 form, for 1836, 1-149th of the whole number, and for 1837, 1-139th—the mean, 1-144th.

"If now we multiply by 144 the sum of 2,712 hernias accruing from the males of from 20 to 21 years, in a gene ral population of ten millions of souls, we shall have the approximative sum total of ruptures affecting the male portion of this population, that is to say, five millions of men will offer very probably a mass of 390,528 affected with hernia, a little more than one-thirteenth.

"The five millions of females, having one-fourth only of the number of ruptures which afflict the other sex, will count only 97,382 subjects of rupture, or about 1: 51.

"And lastly the total number of ruptures amounting to 488,360, will be found to the general population in the ratio of 1 to 20.5; that is to say, between 1: 20 and 1: 21."

This enormous difference of the ratio of hernia to the population at 20 years and to the general population, leads our author to consider this question:

5. *The ratio of hernia to the population at different ages.* This is studied in reference to the male population only; the smaller proportion of ruptures in the other sex, giving to the calculation for females less interest. To find the proportion of hernia during the first year of existence:

Five millions of males give, at this age, 153,627 souls. The tables for 1836 and 1837, united, give, as we have already seen, 1: 52.39 for the ratio of hernia at this age to the whole number. Dividing now, by this quantity, the whole number of ruptures (390,528) furnished by the entire male population, and we have the number of ruptures furnished by 153.627

individuals one year old, viz: 7.453. The proportion is 1: 20.67.

In repeating this operation, owing to the manner in which our author has divided the duration of life into periods, a slight inaccuracy occurs, which he thinks it sufficient to point out. "For example, by attributing in mass a certain number of ruptures to the period from 40 to 50, and by supposing them equally distributed upon each of the ten years composing this period, it is evident that they must be reputed less frequent from 40 to 41, than from 49 to 50, inasmuch as the population is less for the latter than for the former year." Bearing this in mind and repeating the operation for all the periods admitted, we have the following results:

"From birth to 1 year the proportion of ruptures to male subjects is - - - - - - 1: 20.67

| | |
|---|---|
| From 1 to 2 years | 1: 29.09 |
| 2 to 3 " | 1: 36.87 |
| 3 to 4 " | 1: 55.64 |
| 4 to 5 " | 1: 59.72 |
| 5 to 13 " | 1: 77.31 |
| 13 to 20 " | 1: 41.72 |
| 20 to 28 " | 1: 30.74 |
| 28 to 30 " | 1: 20.23 |
| 30 to 35 " | 1: 16.58 |
| 35 to 40 " | 1: 8.41 |
| 40 to 50 " | 1: 8.37 |
| 50 to 60 " | 1: 5.54 |
| 60 to 70 " | 1: 4.37 |
| 70 to 75 " | 1: 3.27 |
| 75 to 80 " | 1: 3.74 |

"There is, if I am not deceived, a powerful interest in thus following from age to age, the ascending or descending progression of hernia, compared to the population. Although pretty frequent in the first year, we see how far it is from justifying the conjecture of Camper, who, in 70 dead male infants, having 7 times found one or indeed both of the testicles still in the abdomen, and 56 times the inguinal canal open, upon one or both sides, concluded that hardly one-ninth of new-born male infants escaped hernia. This is substituting induction for fact, a logical fault, of which works of the XVIII. century show too many examples.

"After the first year, the proportion declines rapidly, so that, allowing for all cures which may take place, the conclusion is inevitable, that the mortality is nearly four times greater from birth to the 13th year, in subjects with hernia than in others. To avoid this conclusion, shall we admit that two-thirds of the ruptures are cured at an early age? The proportion, from what I have seen, appears to me greatly exaggerated; and yet it would not be sufficient, for a certain number of ruptures are developed at the ages of 2, 3, 4 and beyond, which would supply the losses caused by death in hernious subjects of the first year, and which compel us to admit that the mortality is much greater than it appears, or the cures far more numerous.

"After 13 and up to 75, the production of hernia is so active, so out of all proportion with the losses this peculiar population can sustain, that we can draw nothing from the preceding table touching the question of the greatest mortality. But passed 75, our figures will determine the question; supposing no new hernias supervene, the proportion of the preceding period would continue the same; so far from this, the number of ruptures, although constantly reinforced, diminishes more rapidly than the ordinary population. Here no radical cure can be alleged; death alone can commit such havoc. Nor will strangulations account for this increased mortality; these, very rare at any age, are still more so among the old, and offer besides less danger than in adults. Before all things else, I refer it to the inconveniences resulting from the hernia itself, which in the end alters the digestive powers, and thus precipitates the loss of the vital forces."

After the 80th year, the annual decrease of hernia is thus presented by Malgaigne:

From 80 to 83 the proportion to the rest of the population is - - - - - - 1: 14.41
From 83 to 86 - - - 1: 24.93
From 86 to 98 - - - 1: 35.36

But there are two sources of error in the calculations thus presented. From the tables of Villermé, it appears that the general mortality is greater among the poor, than in the other classes. Thus, of ten thousand individuals there remained

| | Rich Departments. | Poor Departments. |
|---|---|---|
| At 60 years - - | 3,127 - - | 2,196 |
| 80 - - | 697 - - | 380 |
| 90 - - | 82 - - | 53 |
| 100 - - | 1 - - | 1 |

Hence it is to these quantities rather than those derived from the law of population that the number of ruptures must be compared.

But besides the mortality which weighs upon the indigent population of Paris, there is another source of diminution, entrance into the public hospitals; after the age of 79, all the destitute have a right to enter into the two hospitals devoted to old age, Bicêtre for males, Saltpétriére for females. It was necessary then to have the exact number really existing at the time of our author's researches. This is furnished by the numerical census of the class, published triennially by the hospital administration. Unfortunately, however, in giving the ages, they have confounded the sexes, and confined themselves to heads of families. Still, taking the number of these, for the years 1836 –'37, as approaching at least pretty near that of all the poor in the latter periods of life, and comparing it with the number of ruptures at corresponding ages, observed at the central bureau (see table at p. 320), we have:

| | | | |
|---|---|---|---|
| For 10,493 paupers, | from 60 to 75, | 1,224 hernia, | 1: 8.57 |
| 2,070 " | " 75 to 80, | 155 " | 1: 13.35 |
| 1,041 " | " 80 to 90, | 41 " | 1: 25.39 |
| 35 " | " 90 to 100, | 1 " | 1: 35. |

These must not be thought the only ruptures furnished by this indigent population. But, continues the author:

"What I desire to establish, and what flows unanimously and invincibly from all these calculations, is, that after the age of 75, the population affected with hernia disappears more rapidly than the ordinary population, four times more quickly, for example, for the period from 80 to 90 years, even taking no account of the new ruptures that occur and partly fill the vacancies made by death in subjects of the preceding period."

6. *The proportion of hernia in the indigent and easy classes.*

Few large cities present, in so decided a manner as Paris, a population divided into a laboring and necessitous portion, and a rich and fashionable portion, each inhabiting its own quarter, and between the two extremes a third, the wealthy and commercial quarter, where wealth and luxury dwell side by side with the most squalid poverty and wretchedness. By comparing the three quarters together, for a series of years, it would seem possible therefore to ascertain what influence, if any, these circumstances have upon the production of hernia.

The first attempt of the kind was made by Richerand, who noticed that among the conscripts for the years XI. and XII., two districts of the city, St. Antoine and St. Marcel, offered more hernia than the others. He explained this result "by the kind of labor to which the inhabitants were devoted," that is to say, "the exercise of mechanical occupations."

In the work of Chabrol, *Recherches statistiques, &c.*, already referred to, our author finds valuable details touching this point; three tables, in which are enumerated the paupers relieved in the twelve districts (*arrondissements*) of Paris, during the years 1821, 1822, and 1823. These show that the greatest number of poor belongs to the sixth, eighth, ninth, tenth and twelfth districts; the least to the first, third and fourth; whilst the middle place is held by the second, fifth, seventh and eleventh.

Comparing now the sum of hernia produced by these districts for the male population of 20 and 21, and for the seven years from 1816 to 1823 (we omit the calculations for each district) we have the following aggregate result:

| | | |
|---|---|---|
| "POOR DISTRICTS. | | |
| "Total examined 4,030, | 142 hernias, | 1: 28.38 |
| "RICH DISTRICTS. | | |
| "Total examined 1,604, | 43 hernias, | 1: 37.30 |
| "EASY DISTRICTS. | | |
| "Total examined 2,913, | 76 hernias, | 1: 38.32" |

The results for the *rich* and *easy* districts are nearly the same, the mean proportion being 1:38. On the other hand, the *pauper* districts exhibit a proportion much greater than the general mean, which, calculated for Paris only, in the years indicated, is 1: 32.86. This shows, also, that hernia is not so frequent in the capital as in the liberties.

"We may say then, in general, that the richer classes are a little less subject to hernia than the pauper class, save the general influences, such as climate, race, and mode of life."

7. The last and not the least interesting point of this inquiry, is, *The relative frequency of hernia in the different parts of France.*

"We have seen that the general proportion, disclosed by the tables of recruits, is the same for Paris and for all France; and this conformity will appear less surprising to those who consider that Paris has not, so to speak, a proper population; that the individuals born in its circumference belong, for the most part, to families from the various provinces; insomuch that we may say of it what the Romans said of Rome, it is an epitome of the whole empire. But out of Paris, these different provinces of which France is composed, do they present the same results? Are ruptures as frequent in the north as in the south, in the mountains and valleys, among the people of Roman and Gallic origin, and among those mingled with foreign blood?"

The War office again, in its returns of recruits for each department of the kingdom, furnishes the materials to elucidate these questions. Until the year 1836, however, none of these reports were published in sufficient detail. Aware of the insufficiency of the data, it was not until after analysing the tables of that year for each department, as he had formerly analyzed those for the whole kingdom, and finding striking and remarkable differences, that he began to feel confidence in the results. Subsequently the tables for 1837, subjected to the same process, and carefully compared with the former, with some partial exceptions verified all these results.

From the mean of these two years he proceeds to develope certain considerations, some of which we shall notice. A map is also given—a map of "*hernious France*"—showing at a glance where the disease is most common. This map is a curious thing in its way, and we are sorry we cannot reproduce it; however, what follows will be sufficiently intelligible with any good map.

We may state here that the general proportion for the departments as deduced from these last researches, is as follows:

| | |
|---|---|
| For 1836, - - - - - | 1: 29.34 |
| For 1837, - - - - - | 1: 33.42 |

Giving a mean term of 1: 31.38. The mean term heretofore deduced, the reader will bear in mind, is 1: 32.

As might be expected, the greatest variety is observed in the amount of hernia presented by the departments. Whilst some fall as low as 1 in 145, others rise as high as 1 in 13. The contrast is marked, the transition sudden, conterminous departments presenting the wide disproportion of 1: 16 and 1: 45, 1: 58 and 1: 116, &c. Hence those where ruptures abound do not rank in well-defined zones, but are found at all points of the compass. Nevertheless it is remarked that they form a large uninterrupted series, cutting France from north to south and from east to west, or from the department of the North to that of the Lower Pyrenees. Taking the 46th degree of latitude as the line of division between northern and southern France, the latter presents ten departments with hernia out of 36, whilst the former counts 22 out of 49. In northern France is a compact mass of 13 departments grouped together, with extensions towards the south and east. In the southern division is found a pretty compact group of five, continued towards the Lower Pyrenees; another small group of three on the side of the Eastern Pyrenees; and in the extreme south-east, the department of Var. It is noted as something curious that hernia is common in the five principal angles of France, viz.: the departments of the North, the Upper Rhine, Lower and Eastern Pyrenees, and of Var.

The opinion that the dwellers among mountains are more subject to hernia than the inhabitants of plains or valleys, is almost universal. The contrary would at first view seem to be established by these researches. The valley of the Seine, that of the Loire, and the Garonne, all present departments abounding in hernia. But so many exceptions occur to this, so many instances of departments distant from mountains falling below the general mean, that without denying the influence of a habitation in mountainous or level districts, Malgaigne thinks other causes must be recognized, whose influence is perhaps greater.

He had shown by previous researches that hernia was more frequent in individuals of high stature than those of medium height. This, too, seems at first to be confirmed by the different proportion of ruptures in northern and southern France. Statistical writers have indeed called the former, France of great stature—*la France des grandes tailles*—and it is evident that ruptures are more common there. Yet particular facts are altogether opposed to this; in the departments of Doubs, Jura, Somme, and Moselle, which send recruits of the finest stature, hernia is rare; whilst Allier, Charente, and Vendeé, where the average stature is low, present hernia in greater proportion than most other departments.

"Thus high stature, like the habitation of plains, establishes a general predisposition to hernia, but within certain limits, and frequently opposed by other conditions which must be sought elsewhere."

To the north-west lie a mass of departments grouped along the littoral confines of the empire, where hernia is infrequent. These are the five departments of ancient Britany, where the proportion varies from 1:58 to 1:116; the two departments of Maine, proportion 1:49 to 1:58; three of Normandy, which give 1:36 to 1:81; and far to the north two others, 1:65 to 1:145. Here occurs one of those sudden transitions to which reference has been made. The three departments of Poitou, conterminous with Brittany, exhibit a proportion of 1:16 to 1:27; below Poitou are three others, which go as high as 1:15 and 1:14. Eastwardly the increase is more gradual; but far to the north is Pas-de-Calais, 1:145, in contrast with the department of the North, 1:16. These strong contrasts, occurring in both years, are to be ascribed to something besides chance.

"It is interesting to observe here that ruptures suddenly become very rare in all the zone where the culture of the vine ceases, and where cider is the general drink. In the department of the North, the population is rich, the cities large, and the vine does not grow; all conditions which seem to favor a less production of hernia. The result is quite the reverse; must it be charged to the general use of beer? I ask a question, rather than give a solution."*

In the southeast, lie 26 departments, which exhibit a variable proportion, though always below the mean; they include the greater part of Languedoc, Auvergne, and Provence, with the whole of March, Limousin, Lyons, and Dauphiny; their continuity is interrupted by four others, whose proportion is above the mean. The most general character of all these departments, is that they are mountainous. A large part of their population make habitual use of oil, a circumstance which enables us to test the opinion advanced by some writers, that oleaginous aliment † favors the production of hernia. The northern limit of the olive, as assigned by Malte-Brun, includes nearly one-half of these departments. With three exceptions, the olive region is below the mean propor-

---

* Apropos to this; Lawrence quotes a passage from Soemmering, who thought potatoes favored the formation of hernia, and another from Blumenbach, who assigns milk as a cause of the great prevalence of ruptures among the Swiss and Dutch. He then remarks with a touch of humor: "It seems strange that a single Irishman should escape the united operation of the milk and potatoes." If to these be added the beer, the Dutch and English would fare quite as badly.

† Train-oil, for example. Hence your Esquimaux would be one immense hernia—unless saved by Liebig's theory.

tion; and the contiguous departments, where the consumption of oil is equal, are among those which, of the whole kingdom, present the smallest number of ruptures.

Lastly, to the northeast, are Burgundy, Franche-Comté, Lorraine, a part of Alsace and Champagne, comprising 13 departments, where the proportion varies from 1:33 to 1:82. Two, and perhaps three exceptions occur, in departments which equal or exceed the general mean.

"To what shall this exemption, for the northeastern part of France, be attributed? Nearly all the departments are occupied by mountains; however, Moselle and Meurthe are quite level; and the former especially offers less hernia than Manche and the Vosges. No doubt it would be rash, with the results of only two years, to launch into conjectures that might be falsified by succeeding years; and all this can pass but for a first glance cast upon an unknown domain, to reveal its existence and call thither new investigators. But I cannot conceal the impression made upon me by these first researches, as the most general consequence; which is, that, if the habitation of mountains and large cities, and the use of certain aliments, seem to defend the population against a too frequent occurrence of hernia, it is to the difference of races that must be ascribed the difference of the proportions pointed out. The vast territory of actual France has experienced, perhaps more than any other portion of Europe, the influence of numerous imigrations. The race, probably the most ancient of the soil, shut up in the Armorican peninsula, and recognizable still by its aspect and its language, is that of the Cymri, the population of ancient Brittany, less subject than any other to the development of hernia. There especially where it is preserved the most pure, the proportion of these infirmities is exceeding small; it increases perceptibly in the sole department where industry and commerce have called foreigners. Maine and Anjou, nearer the centre of the empire of civilization, with a more mixed race, already contrast with Brittany by the number of their ruptures. Something similar is seen in Normandy, where the old population has been replaced, or intermixed with the Danes; there also the department of the Lower Seine, opened by commerce to men of all races, seems to have lost its primitive national vigor. Lastly, it is difficult not to be struck with the contrast between the people of Picardy and Artois on the one hand, and those of the department of the North, on the other, where the Belgic or Flemish race predominates.

"Look now through the northeast; Lorraine, Burgundy, Franche-Comté, will show you the Gallic blood mingled with the Germanic; in the southeast, Lyons and the Dauphin have similar origins; Provence unites in its modern population the descendants of the Ligurians, Greeks, Romans, and Visigoths; and lastly Aquitaine belongs to the Iberian race. There remain at the centre, the families of the Gallic or Gallo-Roman race, better preserved from invasions than the others by their position. This is ancient France, and almost to this day the France of Charles VII., constituted chiefly of Champagne, Isle-of-France, Orleans, Touraine, Poitou, &c., where ruptures are seen to predominate. To the influence of the race, add now, if you will, the influence of the soil inhabited for ages, plains or mountains, then perhaps the influence, less energetic doubtless but always perceptible, of certain aliments; and you have, if I do not deceive myself, the elements necessary to elucidate this capital fact; the differences of the predisposition to hernia in the various provinces of France. Be the explanation what it may, the fact itself is unquestionable; and this difference exceeding the proportion of 4 to 1 in conterminous provinces, as in Brittany and Poitou, is it astonishing that the statistics of hernia in England, Holland, and Belgium, even when most worthy of confidence, give results different from ours?"

Malgaigne, in conclusion, thus meets an objection which these last researches may suggest against the first:

"For, if the rich are less subject to hernia than the poor, the citizen less than the peasant, the mountaineer less than the dweller in the plain, and certain races less than others, is not all that I have elaborated upon the relations of hernia to population, sex and age, again called in question?

"But it must be considered that for the proportion from the age of 20 to 21 years, I have made my calçulations for the whole of France;

That these calculations were found just for Paris in particular, which is an epitome of France;

And that for the other proportions relative to the sexes and the different ages, the population of Paris, which furnished me with the elements, is in reality the one which best represents that of France." C.

